# Validity and Reliability of Knowledge, Attitude and Behavior Assessment Tool Among Vulnerable Women Concerning Sexually Transmitted Diseases 

**Published:** 2016-03

**Authors:** Zahra Boroumandfar, Farzin Khorvash, Katayoun Taeri, Mahrdad Salehi, Ghasem Yadegarfar

**Affiliations:** 1 Infectious Diseases and Tropical Medicine Research Center, Isfahan University of Medical Sciences, Isfahan, Iran; 2 Nosocomial Infection Research Center, Department of Infectious Diseases, Medical School, Isfahan University of Medical Sciences, Isfahan, Iran; 3 Behavioral Disease Council Center, Isfahan University of Medical Sciences, Isfahan, Iran; 4 Psychosomatic Research Center, Isfahan University of Medical Sciences, Isfahan, Iran; 5 Department of Epidemiology and Biostatistics, School of Health, and Heart Failure Research Center, Isfahan University of Medical Sciences, Isfahan, Iran

**Keywords:** Sexually Transmitted Diseases, Knowledge, Attitude, Content Validity, Reliability

## Abstract

**Objective:** The study aimed to design and evaluate the content and face validity, and reliability of knowledge, attitude, and behavior questionnaire on preventive behaviors among vulnerable women concerning sexually transmitted diseases (STDs).

**Materials and methods:** This cross-sectional study was carried out in two phases of an action research. In the first phase, to explain STDs preventive domains, 20 semi- structured interviews were conducted with the vulnerable women, residing at women prison and women referred to counseling centers. After analyzing content of interviews, three domains were identified: improve their knowledge, modify their attitude and change their behaviors. In the second phase, the questionnaire was designed and tested in a pilot study. Then, its content validity was evaluated. Face validity and reliability of the questionnaire were assessed by test re- test method and Cronbach alpha respectively.

**Results:** Index of content validity in each three domain of the questionnaire (knowledge, attitude and behavior concerning STDs) was obtained over 0.6. Overall content validity index was 0.86 in all three domains of the questionnaire. The Cronbach’s alpha as reliability of questionnaire was 0.80 for knowledge, 0.79 for attitude and 0.85 for behavior.

**Conclusion:** The results showed that the designed questionnaire was a valid and reliable tool to measure knowledge, attitude and behavior of vulnerable women, predisposed to risk of STDs.

## Introduction

STDs impose numerous economic and social challenges to many countries (1). The spread of these diseases is notably various in different countries and there is no clear statistic about vulnerable women. Kazerouni et al (2013) showed that Herpes type II and Chlamydia had prevalence of 9.7% and 9% in Iran respectively (2). Guya et al (2006) reported the prevalence of STDs as 11.3% (3). What reveals the importance of STDs follow up and focusing on such diseases is the existence of vulnerable individuals (4) including addicted women, those having an addicted spouse, the couples with history of drug abuse, the women with several sexual partners, or those cheating on their spouse (5). Several studies clarified the incidence of high-risk behaviors such as drug injection inside or outside prison. It threatens the general population and predisposes them to infections of hepatitis C, B and HIV and AIDS (6). A study in Iran showed that among 2063 injection drug abusers, 76.6% had a sexual intercourse in the prior year. Their use of condoms in their last intercourse was 34.5% in men less than 25 years and 32.6% in those over 25 years. Among prostitutes, these statistics were 59% among those less than 25 years and 52% in those over (5). Despite negative outcomes of high risk behaviors among vulnerable women in Iran, the prevailing silence on STDs and lack of attaining correct information about these diseases make it difficult to follow up their incidence and treatment, especially among women, and are accompanied with some limitations in planning educational programs about STDs (7). Lack of knowledge about protective behaviors among high risk women is another reason for their inappropriate function (8). 

One of the most efficient sections of health education programs is evaluation. The measurement tools, selected to achieve an educational program goals, should be also evaluated (9). To reduce measurement errors, general actions such as designing a proper tool, training human resources and tool standardization are taken (10). The goal of a study on construction and psychometric evaluation of a tool is to provide the evidences showing that the constructed or studied tool is valid and reliable (11). Therefore, in the present study, it was essential to describe the main concepts of this tool containing knowledge, attitude and behavior concerning STDs. As judgment, control and prevention of the behaviors resulting in STDs in Iran are dependent of cultural, social and religion backgrounds, the present study was conducted with goal of designing and evaluation of the content validity ratio (CVR), content validity index (CVI), face validity (FC) and reliability of knowledge, attitude, and behavior questionnaire to measure the concepts, adopted in education of preventive behaviors among vulnerable women concerning STDs.

## Materials and methods

This is a cross-sectional study and is a part of an action research. In the first phase, to explain the indexes for the empowerment of the women, at-risk to STDs, 20 semi- structured interviews were conducted with the vulnerable women who resided in women prison and women referred to counseling centers from April 2013 to September 2014. Inclusion criteria in the first phase were being addicted to stimulants or drugs, prostitution, being on treatment with methadone and the interest to attend the study. Sampling continued until data saturation. After content analysis of the interviews, one of the main themes of empowerment of the women who were predisposed to risk of STDs was “empowerment at the personal level”. This theme contained sub- themes of “knowledge promotion”, “modification of attitude and high risk behaviors resulting in STDs”. Then, to investigate the above-mentioned variables, construction of an associated questionnaire was needed. What seemed essential in designing such a questionnaire was how well the selected items could cover all items, which had essential criteria when entered to the final tool (12). After reviewing the transcripts of the interviews, the primary draft of the questionnaire was designed that contained 30 phrases in domain of knowledge, 24 phrases in domain of attitude, and 24 phrases in domain of behavior concerning STDs. It included the types of transmission ways and care (in domain of knowledge), and women’s belief and interpretation (in domain of attitude) concerning STDs and behavior in prevention, treatment and care. CVR and CVI were adopted as the goal of the present study was to design a specific questionnaire to measure knowledge, attitude and behavior-change concerning STDs in vulnerable women. To evaluate CVR, Lavashi suggested methods were adopted (13). To standardize and validate the questionnaire, a panel of 20 experts assembled that working in vulnerable women counseling and care centers .The experts represented content area expertise in counseling, care, treatment STDs. In addition, some of them had expertise and experience in evidence-based medicine, research design and methodology. Then, three sessions were held with expert panel on the content of the questionnaire, and they were asked to code their comments in a judgment scale with respect to Lavashi model in form of “necessary”, “useful but unnecessary”, and “unnecessary”. Next, the questionnaire was designed based on indications of a group of experts. Based on Lavashe table and the number of evaluators, every item obtaining CVR score more than 49% was considered as “necessary” in significance level of 0.05. Waltez and Basal method was adopted to evaluate the content validity index (CVI) in the way that the experts determined “relevancy”, “clarity” and “simplicity” of each phrase, based on a four- point Likert’s scale. CVI was assessed by dividing the number of experts who scored the items 3 or 4 points to the total number of experts (14). CVI scores over 0.79 were considered appropriate. Face validity of the questionnaire was made based on experts panel suggestions and the suggestions of the women predisposed to risk of STDs on each phrase. Questionnaire Reliability assessment was done by test re- test method and Cronbach alpha. In test re- test method, the questionnaire was filled by 20 vulnerable women in two steps with a ten- day interval through questioning method. Concerning internal consistency (Cronbach alpha), the questionnaire was once filled by 20 vulnerable women through questioning method. In both reliability evaluations, SPSS 18 was adopted to analyze the data. It should be noted that the researcher firstly attained needed permissions from ethics committee of vice- chancellery for health and Isfahan prison organization to access and collect data, and then, after taking participants’ written consents conducted the interviews and completed the questionnaires (in second phase) in a pilot study.

## Results

The results in the first phase showed that participants’ mean age was 28 ± 9 years. Most of participants (55.7%) had a primary education, (88.7%) were homemaker, (60%) got married, (85%) of them had experienced History of prostitution, (90 %) of them had experienced History of addiction, and (85.5%) being on treatment with methadone. In the second phase, mean age and mean work experience of experts panel members were 40 years and 15 years respectively. They had education degrees varying from bachelor’s degree to PhD. Results showed that content validity for all phrase in three domains of the questionnaire (knowledge, attitude and behavior concerning STDs) was over 0.6. Meanwhile, 4 phrase in knowledge measurement questionnaire with validity index of 0.3, 5 phrase in attitude with validity index of 0.4 and 4 phrase in behavior measurement questionnaire with validity index of 0.2 were deleted. CVI was estimated 0.85 for knowledge, 0.83 for attitude and 0.90 for behavior (Tables 1, 2, 3). Face validity of all questionnaires was reviewed, modified and in some cases, deleted by expert panel and the women, predisposed to at risk of STDs. In cases where vulnerable women’s views on STDs (attained through holding 4 sessions with them) were different from those of the expert panel, it was tried to consider vulnerable women’s views. For instance, phrases such as “poverty (financial need)” may lead women to prostitution”, “addiction may result in women’s prostitution”, “lack of spouse’s affection may result in women’s infidelity”, “when my spouse suffers from infection and inflammation of genital area, I avoid having sex with him” were indicated and emphasized by vulnerable women while they were not indicated as “necessary” by the expert panel. Reliability of the questionnaire was not only assessed by Cronbach alpha in knowledge (0.80), attitude (0.79) and behavior (0.85) measurements but by test re- test and Pearson correlation coefficient as well (r = 0.87).

## Discussion

Present study is among the first studies on designing a reliable and valid tool to investigate the level of knowledge, attitude and behavior of vulnerable women, predisposed to at risk of STDs in Iran. One of its advantages is that designing of the phrases was made by referring to the transcripts of deep and semi- structured interviews with addicted women having high risk sexual intercourse and being predisposed to at risk of STD. Then, qualitative content analysis was extracted and designed in accordance with vulnerable women’s request for promotion of knowledge and a behavior change to protective behaviors against STDs. WHO also reported women’s and girls’ need assessment, especially in field of hygiene and health, as a priority and a principle for function (15, 16). Another advantage of this tool is that through it, level of knowledge, concerns and sexually high risk behaviors resulting in STDs can be investigated in vaster spam of vulnerable women including prostitutes, drug abusers and those with sexually high-risk behaviors spouses and addiction as these women are almost similar in one aspect (sexually high -risk behaviors). 

**Table 1:** Measurement of knowledge of STDs

		**CVI**	**CVR**
**1**	**Vagina is a part of women’s external genital system.**	**0.80**	**0.60**
**2**	**Urethra and semen outlet are the same in men.**	**0.80**	**0.60**
**3**	**High odorless secretions in mid- menstruation period is normal.**	**0.80**	**0.70**
**4**	**Women may be involved in genital infection after intercourse.**	**0.86.6**	**0.90**
**5**	**Venereal diseases may have no signs in men**	**0.83**	**0.80**
**6**	**Venereal diseases are usually transmitted by men’s and women’s dirty hands.**	**0.86.6**	**0.90**
**7**	**Drug abuse can increase the risk of STDs and high risk sexual behavior.**	**0.83**	**0.80**
**8**	**Diagnosis of venereal diseases in women is made by genital system examination.**	**0.90**	**0.80**
**9**	**Venereal diseases need complete treatment in men and women.**	**0.86.6**	**0.80**
**10**	**Gonorrhea can be also transmitted by shaking hands.**	**0.83**	**0.80**
**11**	**HIV can be transmitted by kissing.**	**0.83**	**0.90**
**12**	**Risk of STDs is less in anal sex.**	**0.90**	**0.80**
**13**	**Venereal diseases can be also transmitted through oral sex.**	**0.83**	**0.80**
**14**	**Morning secretions from penis is normal.**	**0.86.6**	**0.80**
**15**	**Genital system warts are a sign of STDs.**	**0.93**	**0.90**
**16**	**Women’s high genital system secretions and itchiness can be a sign of STDs.**	**0.83**	**0.60**
**17**	**STDs cause pain and urinary frequency during intercourse.**	**0.86.6**	**0.60**
**18**	**AIDS can be transmitted through intercourse.**	**0.86.6**	**0.90**
**19**	**Gonorrhea can be cured completely after treatment.**	**0.83**	**0.80**
**20**	**Hepatitis B is also STDs.**	**0.83**	**0.90**
**21**	**During treatment of venereal diseases, a condom should be used in intercourse.**	**0.80**	**0.80**
**22**	**Treatment STDs in couples should be administrated concurrently.**	**0.86.6**	**0.90**
**23**	**Rinsing the genital system after a rape can prevent STDs.**	**0.80**	**0.60**
**24**	**After a rape, an emergency contraception pill should be taken.**	**0.90**	**0.90**
**25**	**Female condoms are more efficient in prevention of STDs, compared to male condom.**	**0.83**	**0.90**
**26**	**Groin lump can be a sign of STDs.**	**0.86.6**	**0.70**
**Total**		**0.84.7**	**0.76**

**Table 2:** Measurement of attitude concerning STDs

		CVI	CVR
**1**	**Contamination with some STDs can never be treated and remains life long.**	**0.80**	**0.60**
**2**	**Talking about women’s sexual relation leads to their corruption.**	**0.83**	**0.60**
**3**	**Condom can absolutely prevent STDs.**	**0.83**	**0.90**
**4**	**HIV test is a shame.**	**0.83**	**0.80**
**5**	**Through use of a condom, I am almost sure not to have any problems with STDs in any type of intercourse.**	**0.83**	**0.80**
**6**	**Only sexual relation with a prostitute contaminates men with STDs.**	**0.83**	**0.90**
**7**	**Poverty (financial problems) may force women to prostitution.**	**0.80**	**0.70**
**8**	**I will have less fear of STDs by receiving information about them.**	**0.86.6**	**0.60**
**9**	**Women will be safe against venereal diseases by following personal hygiene.**	**0.86.6**	**0.60**
**10**	**If a woman suffers from STDs, she should inform her sexual partner or spouse.**	**0.83**	**0.90**
**11**	**STDs are not life threatening and need no follow up.**	**0.83**	**0.90**
**12**	**Addicted women are indifferent about their venereal diseases.**	**0.80**	**0.90**
**13**	**Guilty men and women are involved STDs.**	**0.83**	**0.60**
**14**	**Women’s examination is not a shame.**	**0.86.6**	**0.60**
**15**	**Contraceptives also prevent STDs.**	**0.90**	**0.70**
**16**	**Judgment of health providers on my probable STDs prevents of my referring for treatment.**	**0.79.6**	**0.60**
**17**	**Due to the fear of not keeping my STDs secret confidential, I avoid referring to health care centers.**	**0.80**	**0.80**
**18**	**Lack of affection from the spouse may lead to wife’s infidelity.**	**0.86.6**	**0.80**
**19**	**Addiction may lead to prostitution.**	**0.83**	**0.70**
**Total**		**0.85**	**0.73**

Niknamy et al (2004), in a study on the effect of health education on prevention of AIDS among the spouses of the self – reported addicts in Kermanhah, Iran, investigated the content validity of a researcher made questionnaire based on HIV texts and merely measured studied women’s knowledge about HIV (17). In study of Teimouri (2011), on prevalence of high- risk behaviors and the sexually transmitted infections in women referring to addiction cessation centers, the researchers designed a questionnaire based on texts, articles and polls among academic members (18). Among other actions, taken to increase validity in the present study, was the cooperation of tool making experts who were aware of vulnerable women’s high-risk behaviors in care and treatment of STDs. On the other hand, one of the most valid content validity methods (evaluate of CVR and CVI) was adopted in the present study. Our obtained final content validity rate of 0.75 shows that the selected phrases in the present questionnaire could acquire the needed criteria to be entered to the tool. Its final content validity index (0.86) shows that the items had high scales of simplicity, relevancy and clarity. In fact, content validity index shows wholeness of the judgments, associated to validity or applicability of the model, test or final tool (19). In study of Mona Larki (2014), content validity ratio was reported 0.89 and content validity index was 0.98 (20), which is somehow consistent with the present study. Results showed that our obtained reliability indexes in both methods were consistent with the proper amounts, reported in text books of statistics. Cronbach alpha = 0.7 has usually been reported as an acceptable level for determination of reliability. Cheri Cupmna reported correlation coefficient and Cronbach alpha of 0.49 and 0.81 in his scale of beliefs in AIDS prevention respectively (21). In the present study, overall Pearson correlation index of the questionnaire was 0.81 revealing the acceptability and appropriateness of the indexes obtained from questionnaire reliability and proves its applicability to determine knowledge, attitude and behavior of the vulnerable women concerning STDs in Iranian society or any other similar society.

## Conclusion

Designing a data collection tool and determination of its validity and reliability are a costly and time-consuming step. If there are tools for various subjects whose validity and reliability are measured and confirmed, they can speed up the trend of research and lower the research costs.

**Table 3:** Measurement of behavior concerning STDs

		CVI	CVR
**1**	**In case of burning and itchiness after intercourse, I refer for treatment.**	**0.86.6**	**0.70**
**2**	**In case of observing and touching a wart in my genital system, I refer for treatment.**	**0.90**	**0.90**
**3**	**I encourage my sexual partner to go for treatment if he has genital system wart.**	**0.90**	**0.70**
**4**	**I encourage my spouse to go for treatment if he has genital system wart.**	**0.90**	**0.70**
**5**	**I rinse my genital system before and after intercourse.**	**0.86.6**	**0.60**
**6**	**I use women condom if my sexual partner is not interested in using a condom.**	**0.86.6**	**0.80**
**7**	**I use female condom if my spouse is not interested in using a condom.**	**0.83**	**0.70**
**8**	**In case of STDs, I take medication concurrently with my spouse/ sexual partner.**	**0.86.6**	**0.90**
**9**	**I wear condom before starting sex.**	**0.83**	**0.70**
**10**	**After once treating infection and inflammation of genital system, I go on with follow ups**	**0.86.6**	**0.80**
**11**	**I avoid having sex when my spouse suffers from genital system infection, edema, and inflammation.**	**0.86.6**	**0.70**
**12**	**I avoid having sex when my sexual partner suffers from genital system infection, edema, and inflammation.**	**0.86.6**	**0.70**
**13**	**I take part in group- sex.**	**0.83**	**0.80**
**14**	**I take drugs when having sex.**	**0.86.6**	**0.80**
**15**	**I decided to lower the number of my sexual partners to protect myself STDs.**	**0.83**	**0.70**
**16**	**I avoid having oral sex to prevent STDs.**	**0.86.6**	**0.90**
**17**	**I avoid having anal sex to prevent STDs.**	**0.86.6**	**0.90**
**18**	**I can say no to unprotected high-risk sex.**	**0.90**	**0.90**
**19**	**I resist when offered more money for unprotected sex.**	**0.83**	**0.90**
**20**	**I use condoms when having sex with my Sighe husband (temporary marriage).**	**0.80**	**0.80**
**Total**		**0.86.05**	**0.79**

In the other words, they can prevent repetition in research with regard to the cultural and religious dimensions of societies similar to Iran. The researchers of the present study made their all effort in this direction 0.8.
